# Fluoxetine Reduces Hyperglycemia‐Induced Facilitation of Fear Memory in Mice

**DOI:** 10.1002/brb3.71206

**Published:** 2026-03-09

**Authors:** Kayla R. Gilley‐Connor, Laura E. Kusumo, Grace M. Hall, Matthew J. Folh, Elisabeth G. Vichaya

**Affiliations:** ^1^ Department of Psychology and Neuroscience Baylor University Waco Texas USA; ^2^ Department of Biology and Chemistry Liberty University Lynchburg Virginia USA; ^3^ Department of Neuroscience & Experimental Therapeutics Texas A&M University Bryan Texas USA

**Keywords:** depression, diabetes, negative valence bias, streptozotocin

## Abstract

**Introduction:**

Although it is well established that people with diabetes are at an increased risk of developing neuropsychiatric disorders, including depression and anxiety, the mechanisms that mediate this relationship are not fully understood. The use of preclinical models can be helpful in the investigation of mechanisms. For example, studies have shown that chronic hyperglycemia (HG), induced by administration of streptozotocin (STZ) to mice, induces neuroinflammation and depressive‐like behaviors in classic tests of antidepressant efficacy (e.g., forced swim test, splash test). However, there is a need to establish more specific assessments of affective dysfunction, including assessment of changes within the negative valeence systems. It has been previously reported that HG rodents show increased susceptibility to fear conditioning, potentially through increased salience of negative stimuli. Based on this, we sought to determine whether this enhanced fear memory is sensitive to fluoxetine (FLX), a commonly used antidepressant.

**Methods:**

Male C57BL/6J mice were administered 50 mg/kg STZ per day for five consecutive days or an equal volume of citrate buffer to induce HG. Four weeks after HG induction, a time at which behavioral changes are well established, mice started daily treatment with 10 mg/kg FLX or saline vehicle. After approximately 2 weeks of treatment, mice were evaluated for activity (i.e., marble burying and open field) and memory within the fear conditioning paradigm.

**Results:**

As previously reported, mice with HG showed reduced marble burying, reduced open field activity, and increased freezing in the conditioning context. Although FLX treatment did not reverse HG‐induced burying or activity effects, it did reverse HG‐facilitated freezing. This effect was independent of changes in HG‐induced hippocampal *Tnf* expression.

**Conclusions:**

This provides support for the utilization of the fear conditioning task to understand HG‐associated changes in the negative valence systems. It also provides additional confirmatory data indicating that the basal lateral amygdala is sensitive to HG‐induced dysfunction.

## Graphical abstract

Hyperglycemia (HG) enhances fear memory in mice, potentially reflecting negative valence bias rather than cognitive enhancement. Fluoxetine (FLX) prevented HG‐induced facilitation of fear conditioning independent of locomotor activity or hippocampal *Tnf* expression changes. These findings support using fear conditioning to assess HG‐associated affective alterations in negative valence systems.

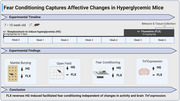

## 1 Introduction

Diabetes mellitus (DM) is a multifaceted metabolic disorder associated with elevated blood glucose levels. It is increasingly recognized that DM is associated with increased rates of neuropsychiatric disorders, including depression and anxiety (Anderson et al. 2001; Farooqi et al. 2022; Mersha et al. 2022). Although stress from disease management is likely to contribute to this elevated risk, disease‐associated factors directly increase this risk by impacting brain health. For example, using a murine model of chronic hyperglycemia (HG), our lab has previously demonstrated that elevated blood sugar levels are associated with neuroinflammation and a variety of behavioral changes associated with depression, including increased immobility in the forced swim task and reduced grooming in the splash test (McCready et al. 2023). While tasks such as these have been shown to respond to antidepressants (Franceschelli et al. 2015; Kryst et al. 2022; Mutlu et al. 2012), they are less suitable for distinguishing specific neural circuits and brain regions impacted in models of depression (Sewell et al. 2021). Therefore, it is important to identify alternative assessments of affective dysfunction.

The streptozotocin (STZ)‐induced model of HG is commonly used to evaluate affective dysfunction (Bampi et al. 2020; McCready et al. 2023; Sakurai et al. 2021). STZ induces HG by preferentially damaging insulin‐producing pancreatic β‐cells. In evaluating the literature using the STZ model, an assessment with the potential to facilitate circuit‐level understanding of STZ‐induced changes is the fear conditioning (FC) task. There is evidence that STZ‐treated rodents demonstrate increased freezing responses during FC (Gambeta et al. 2016; Ikeda et al. 2015). Although FC is generally used to evaluate cognitive deficits, such that decreased freezing is associated with compromised amygdala‐based memory, the facilitated learning demonstrated through increased freezing behavior within the STZ model can be reinterpreted as increased salience of aversive stimuli, or a negative valence bias. A negative valence bias is believed to play a role in motivation and major depressive disorder (MDD) (Bigot et al. 2024; Vichaya and Dantzer 2018; X. Yang et al. 2023). Models of stress‐associated depressive‐like behavior, including 15 days of chronic social defeat stress and chemogenetic activation of neurons governing the release of corticotropin‐releasing factor (CRF), have also reported facilitated FC (Azzinnari et al. 2014; Montgomery et al. 2024). This is consistent with a clinical report that indicates that MDD patients showed enhanced acquisition of aversive stimuli and increased plasticity of emotional systems despite decreased plasticity in networks that govern executive function (Nissen et al. 2010). In addition, experimentally induced inflammation has been shown to increase sensitivity to negative stimuli (Aubert and Dantzer 2005; Harrison et al. 2016; Muscatell et al. 2016).

Although facilitation of FC in the STZ model has been associated with various mechanisms, including oxidative damage and glutamate activity (de Lima Silva et al. 2022; de Souza et al. 2019; Ikeda et al. 2021), it has not yet been directly investigated in the context of an antidepressant. Therefore, within the current study, we sought to validate the antidepressant sensitivity of this task by treating hyperglycemic mice with a saline vehicle or fluoxetine (FLX) daily prior to evaluating FC. FLX is a selective serotonin reuptake inhibitor (SSRI) commonly used to treat MDD (Magni et al. 2013), and is a commonly prescribed antidepressant in the context of DM, as it has been shown to help maintain or even improve glycemic control (Zhang et al. 2022). However, the relationship between antidepressant usage and diabetes is complex, with some antidepressants promoting HG and weight gain (Barnard et al. 2013; Kryst et al. 2022; Ma et al. 2011). FLX has also been extensively studied in preclinical mouse models of depressive‐like symptoms (Castagné et al. 2010; Kryst et al. 2022; Mutlu et al. 2012). We hypothesized that hyperglycemic mice would display increased freezing behavior in the context in which they had previously been exposed to shock and that FLX administration would normalize this response. To further characterize the behavioral effects of HG and FLX, we also assessed general activity and marble burying (MB), as these behaviors are also affected in the STZ model of HG. Finally, we sought to determine whether FLX would attenuate HG‐induced neuroinflammation, measured by hippocampal inflammatory cytokine expression.

## 2 Methods

### 2.1 Animals and Experimental Design

In this study, we used male C57BL/6J mice bred in house. They were maintained at 22°C on a 12‐h light/dark cycle with food and water ad libitum, were housed with two to three mice per cage, in ventilated cages with cotton nestlets. They were handled for 5 days prior to the study start. Testing and procedures took place during the light phase (0700–1900 h). All procedures were reviewed and approved by the Baylor University Institutional Animal Care and Use Committee (IACUC protocol number: 1550457).

We used a 2 (STZ vs. buffer) × 2 (FLX vs. saline) factorial design with 10–11 mice per group (*N* = 42). Mice were 7–10 weeks of age at the start of STZ injections (designated Experimental Week 0). FLX administration began at the start of Week 4 and continued daily for 2 weeks through the remainder of the study. Behavioral testing, including MB, open‐field test (OFT), and FC, was conducted during Week 6. Tissue was collected following the completion of behavioral testing. Data were collected across three cohorts of mice.

### 2.2 Drug Treatments and Health Monitoring

STZ (Sigma Aldrich, catalog number S0130) was administered via intraperitoneal (ip) injections at a dose of 50 mg/kg/day for five consecutive days. Vehicle control animals were administered an equal volume of citrate buffer (pH 4.5) (Furman 2021). On Week 4, mice were randomly assigned to either saline or FLX treatment. FLX (Millipore Sigma, catalog number BP797) was administered via ip injections at a dose of 10 mg/kg/day for 2 weeks. This dose has previously been shown to recover neurogenesis and depressive‐like behavior (Hodes et al. 2010; Kamei et al. 2003; Kryst et al. 2022).

Body weight and 4 h‐fasted blood glucose levels were collected prior to STZ (baseline) and monitored regularly. Blood glucose was measured using an AUVON handheld glucometer and test strips following a nick to the tail. HG was defined as a blood glucose level over 250 mg/dL.

### 2.3 Behavioral Testing

#### 2.3.1 Marble Burying

The MB task is often used to measure anxiety‐like or compulsive behavior in mice (Angoa‐Pérez et al. 2013; de Brouwer et al. 2019). Our lab and others have shown that STZ treatment produces a robust deficit in MB (Gazzo et al. 2021; Kusumo et al. 2024; McCready et al. 2023). This behavior may be indicative of reduced motivation and/or exploratory interest. We included this behavioral task due to its robust phenotype in this model. For the task, each mouse was placed in an arena (30 cm × 19 cm × 13 cm) with fresh bedding and 20 marbles (arranged in 5 rows of 4 marbles) for 30 min. Marbles buried with at least two‐thirds coverage by bedding were scored by a rater blinded to the experimental condition.

#### 2.3.2 Open‐Field Test

The OFT has been used to assess exploration in a novel environment, with duration in the center of the field being used to evaluate anxiety‐like behavior (Seibenhener and Wooten 2015). Our prior work has shown that mice show mild decreases in total exploration but no changes in center time (McCready et al. 2023). Mice were placed in a novel plexiglass arena (30 cm × 30 cm × 40 cm) for 5 min, and activity was recorded. Total distance traveled and center duration were determined using Noldus EthoVision XT software (Leesburg, VA, USA).

#### 2.3.3 Contextual FC Test

FC is a classical measure of memory in which mice are exposed to aversive mild foot shocks in a novel environment and tested for memory of that experience by monitoring freezing when returned to the environment. Freezing is a species‐specific defense reaction to fear (Kim and Fanselow 1992). This form of memory involves the hippocampus and amygdala (Kim and Cho 2020; Phillips and LeDoux 1992). Interestingly, while the STZ model of HG frequently reports detriments in cognitive tasks (Revsin et al. 2009; J. Wang et al. 2018), prior research has reported increased freezing behaviors in this particular task (Gambeta et al. 2016; Ikeda et al. 2015).

We utilized Coulborn Instrument chambers (7.5″ × 8″ × 11″) with shock‐grid floors (bars 3.2 mm in diameter spaced 7.9 mm apart). During training (Day 1), mice were allowed to habituate to the chamber for 2 min, and then 3 foot shocks (0.5 mA, 2‐s duration, 1 min apart) were delivered following a tone (2800 Hz, 85 dB, 30 s). Behavior was recorded by an overhead camera within the chamber. Mice were removed from the chamber 1 min after the last shock.

Twenty‐four hours after conditioning (Day 2), mice were tested for fear memory. Mice were returned to the original conditioning chamber in the absence of shock, and freezing behavior was recorded for 3 min. Activity levels were analyzed using Freezeframe 4 software (Actimetrix). Freezing behavior was determined by tracking the duration of immobility bouts (defined as the absence of movement for at least 0.25 s). The percentage of time spent freezing was used as the measure of fear memory.

### 2.4 Tissue Collection and Analysis of Cytokine Expression

The day after completion of behavioral testing, the mice were euthanized by CO_2_ followed by transcardial perfusions with phosphate buffered saline. Hippocampal tissue was collected, immediately snap frozen in liquid nitrogen, and stored at −80°C until processing.

RNA was extracted from the hippocampus using the E.Z.N.A. RNA Isolation Kit II (Omega BioTek, Norcross, GA, USA). cDNA was transcribed using the High‐Capacity cDNA Reverse Transcription Kit (Applied Biosystems by Life Technologies, Grand Island, NY, USA), and quantitative real‐time polymerase chain reaction (qPCR) was carried out in a QuantStudio 6 PCR machine using TaqMan gene expression assays (Applied Biosystems by Life Technologies, Grand Island, NY, USA). We measured *Tnf* (ThermoFisher, catalog number Mm00443258_m1), *ll6* (Mm.PT.58.10005566), and *Il1b* (Mm.PT.58.41616450) mRNA expression within the hippocampus. *Gapdh* (Mm99999915_g1) and *Rplp0* (Mm.PT.58.43894205) were selected as the housekeeping controls. Their average value was used to calculate the ΔCT. Reactions were performed in duplicate, with the fold difference for each gene calculated using the 2^−ΔΔCT^ method.

### 2.5 Statistical Analyses

Data are presented as means ± standard error of the mean (SEM). Two‐way ANOVAs, with a repeated measure as appropriate, were run in IBM SPSS Statistics 29 to evaluate the effects of STZ and FLX treatments. *p*‐values less than 0.05 were considered statistically significant. Effect sizes were computed using partial eta‐squared (*η* ^2^_p_).

## 3 Results

### 3.1 Body Weights and Blood Glucose Levels

**FIGURE 1:**
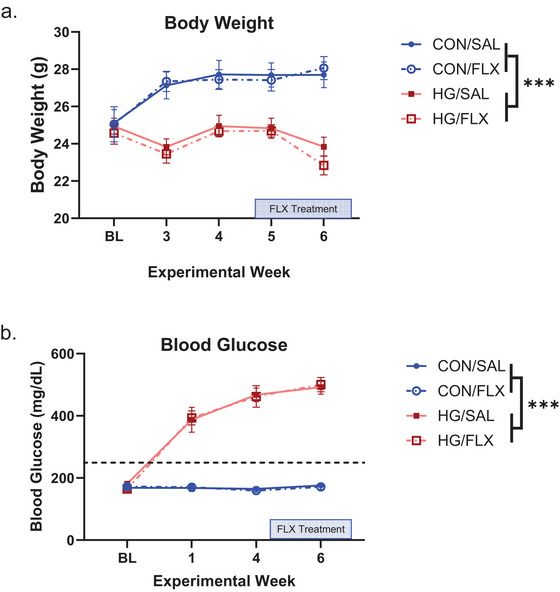
(a) Body weight was measured consistently throughout the study, and HG resulted in consistently lower body weights compared to CON mice. (b) Fasting blood glucose was monitored with the hyperglycemic threshold set at 250 mg/dL; all STZ‐induced HG mice exceeded this threshold postinduction. Data are graphed as means ± SEM; ***p < 0.001. *n* = 10–11 mice per group. CON, control; FLX, fluoxetine; HG, hyperglycemia; Sal, saline; SEM, standard error of the mean.

Mouse body weights were monitored throughout the study to ensure the health of all animals (Figure 1a). As anticipated, there was a significant main effect of STZ treatment *(F* [1, 38] = 28.57, *p *< 0.001, *η* ^2^_p_ = 0.429) such that hyperglycemic mice lost weight over time. FLX treatment did not significantly influence body weights.

Fasting blood glucose was measured throughout the experiment (Figure 1b). There was a significant main effect of STZ treatment *(F* [1, 38] = 497.80, *p *< 0.001, *η* ^2^_p_ = 0.929) such that STZ‐treated mice rapidly developed HG and maintained elevated blood glucose levels throughout the study. FLX treatment did not significantly impact blood glucose levels.

### 3.2 Behavioral Testing

**FIGURE 2:**
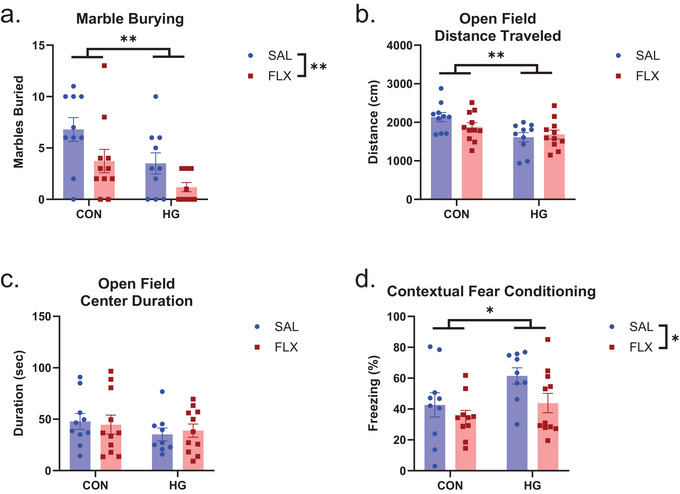
(a) For marble burying, there was a significant main effect of HG and FLX, such that both manipulations reduced the number of marbles buried. (b) Distance travelled in an open field was reduced in HG mice. (c) There were no significant effects of HG or FLX on center duration in the open field. (d) For contextual fear conditioning, HG significantly increased the percent time freezing while FLX reduced freezing time. CON, control; FLX, fluoxetine; HG, hyperglycemia; Sal, saline; SEM, standard error of the mean.*p < 0.05; **p < 0.01. *n* = 10–11 mice per group.

We evaluated MB and OF activity as these behaviors are sensitive to the effects of HG. As anticipated, we observed a significant main effect of HG on MB (*F* [1, 38] = 8.951, *p *< 0.005, *η* ^2^_p_ = 0.191), such that hyperglycemic mice buried significantly fewer marbles. There was also a main effect of FLX treatment (*F* [1, 38] = 7.613, *p *= 0.009, *η* ^2^_p_ = 0.167) with FLX‐treated mice exhibiting reduced MB (Figure 2a). For distance traveled in the OFT (Figure 2b), there was a significant main effect of HG (*F* [1, 38] = 9.373, *p *= 0.004, *η* ^2^_p_ = 0.198) such that HG reduced exploratory behavior in the novel arena. For center duration (Figure 2c), we observed no significant main effects or interactions.

The primary objective of this study was to evaluate the effect of FLX on FC in STZ treated mice (Figure 2d). We measured percent freezing behavior when mice were returned to the context in which they had been previously shocked. Results indicate a significant main effect of HG (*F* [1, 36] = 5.110, *p *= 0.030, *η* ^2^_p_ = 0.124), such that STZ‐induced HG significantly increased the percent freezing. A significant main effect of FLX was also observed (*F* [1, 36] = 4.289, *p *= 0.046, *η* ^2^_p_ = 0.106), whereby FLX treatment significantly reduced percent freezing. Analyses of two mice were excluded in the FCT due to technical difficulties.

### 3.3 Hippocampal Cytokine Expression

**FIGURE 3:**
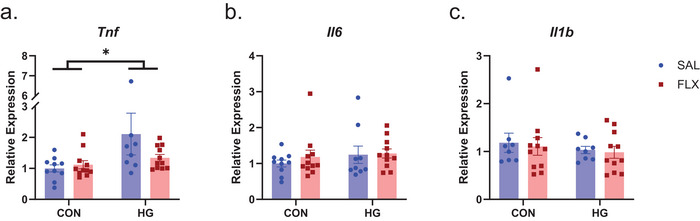
(a) Hippocampal *Tnf* mRNA expression was increased in response to HG; this effect was not significantly attenuated by FLX. (b, c) Neither STZ nor FLX significantly impacted the expression of *Il6* or *Il1b*. Data are graphed as means ± SEM. *p < 0.05, *n* = 8–11 mice/group. CON, control; FLX, fluoxetine; HG, hyperglycemia; Sal, saline; SEM, standard error of the mean.

To determine if the observed effects of FLX in the FC task were mediated by modulation of STZ‐induced neuroinflammation, we evaluated *Tnf, Il6*, and *Il1b* mRNA expression within the hippocampus. There was an effect of HG on *Tnf* expression (*F* [1, 36] = 5.265, *p *= 0.028, *η* ^2^_p_ = 0.128); it was not significantly modulated by FLX (Figure 3a). There was no significant effect of either HG or FLX on the expression levels of *Il6* and *Il1b* (Figure 3b,c).

## 4 Discussion

Within the current manuscript, we aimed to replicate the previously reported facilitation of FC in response to STZ‐induced HG and determine if this effect is responsive to antidepressant treatment. As anticipated, hyperglycemic mice showed increased freezing behavior in the context in which they were exposed to foot shock. In addition, treatment with FLX, a commonly used SSRI, reversed this HG‐induced facilitation. This provides support for the utilization of the FC task to understand HG‐associated affective dysfunction, particularly related to the negative valence systems. It also provides additional confirmatory data indicating that the basal lateral amygdala is sensitive to HG‐induced dysfunction. This effect appears to be independent of neuroinflammation, as FLX did not reverse HG‐induced *Tnf* expression. FLX also did not influence blood glucose and failed to recover activity deficits in the open field and MB tasks, suggesting that these deficits are mediated by distinct mechanisms.

Treatment with FLX was initiated approximately 4 weeks after the induction of HG, as this time has been shown to be sufficient for the development of depressive‐like symptoms (Y. Wang et al. 2021). Hyperglycemic mice displayed the expected lower body weights and elevated blood glucose levels. While daily injections may have induced mild weight loss in the HG mice, this effect was not specifically associated with FLX, as it was noted in both saline and FLX groups. There have been prior reports of effects of FLX on blood glucose in STZ‐treated rats; however, these studies utilized significantly higher doses of FLX and longer treatment windows (Tembhurne and Sakarkar 2011; H. Yang et al. 2020).

We included assessments of MB and open field as we have previously shown these simple tests to be sensitive to STZ‐induced HG (McCready et al. 2023). We observed HG reductions in activity within both tasks; FLX did not recover these HG‐induced behavioral deficits. In fact, we observed that FLX also reduced MB. This effect may be due to the lack of specificity in this task. Increased MB is often considered a measure of anxiety or compulsiveness. In this context, FLX has previously been shown to reduce MB (Arora et al. 2013; X. Li et al. 2006; Nicolas et al. 2006). As such, our data align with those effects; however, it also indicates that the mechanism driving this behavior may be related to the residual symptoms of depression or may be independent of HG‐induced depressive symptoms. These behavioral changes may relate to apathy or lack of exploratory interest. While residual symptoms of depression are not fully characterized and can be challenging to disentangle from the side effects of antidepressants, there is evidence that emotional blunting—associated with apathy—may be a frequent experience of patients being treated with antidepressants (Goodwin et al. 2017).

While FLX did not alleviate HG‐induced behavioral deficits in OFT or MB, FLX did attenuate HG‐induced facilitation of FC. While FC is often used to assess deficits in amygdala‐ or hippocampal‐based learning and memory (Phillips and LeDoux 1992), STZ has been shown to facilitate learning in this task (Gambeta et al. 2016; Ikeda et al. 2015) as has social stress exposure (Azzinnari et al. 2014). Patients with depression have a negative valence bias that appears to be associated with increased activity within the amygdala (Bigot et al. 2024; Dannlowski et al. 2007; Victor et al. 2010). This effect has also been observed within an animal model of depression (Bigot et al. 2024). Further, there are both clinical and preclinical evidence that SSRIs can attenuate this bias (Bigot et al. 2024; Godlewska et al. 2012). As such, we hypothesize that learning is facilitated in the FC task as the shocks are perceived as more aversive and, thereby, the memory of the event is better encoded. This would suggest that FC in this model may be a valid behavioral readout of depression‐associated negative valence bias.

The final aim of this study was to evaluate if FLX was able to reduce HG‐induced brain inflammation. The behavioral consequences of STZ‐induced HG have largely been attributed to elevations in oxidative stress and inflammation within the brain (Bampi et al. 2020; Kotagale et al. 2021; Rahmati et al. 2021; Sakurai et al. 2021; J. Wang et al. 2018). As we have previously reported (McCready et al. 2023), STZ‐induced HG was associated with increased hippocampal *Tnf* mRNA expression without a significant effect on *ll6* and *ll1b* levels. While others have reported that FLX has anti‐inflammatory effects in the context of STZ (Yuan et al. 2019), we did not observe a significant effect of FLX on *Tnf* expression. This difference may relate to the differential timing of treatment; while we started FLX approximately 4 weeks post‐STZ induction of HG, Yuan et al. (2019) started treatment prior to STZ exposure.

Overall, this study supports our hypothesis that HG‐induced facilitation of FC can be reversed by FLX treatment. This supports the utilization of this task to further investigate the mechanisms by which HG impacts the brain and increases susceptibility to depression. It also indicates that future research examining region‐specific changes, including changes within the amygdala, are warranted. There are a few limitations to this study that should be mentioned. Within this study, we only utilized one dose and route of administration. The dose was selected based on prior work showing efficacy in mice on other outcome measures in mouse models of depression (Hodes et al. 2010; Kamei et al. 2003), and the ip route was selected based on evidence of greater efficacy in stress‐induced depression than oral gavage (Q. Li et al. 2022). It is, however, possible that we may have observed effects of FLX on other behavioral outcomes, blood glucose levels, or on *Tnf* expression using different doses and/or routes of administration. An advantage of our selected dosing regimen was that it allowed us to parse the effects in FC from these other changes. Another limitation of this study is that it is unable to rule out the effect of FLX on HG‐induced pain sensitivity. There are mixed findings regarding whether the STZ‐induced model of HG induces neuropathy (Anjaneyulu and Chopra 2004; Sullivan et al. 2007; Tembhurne and Sakarkar 2011). Future studies are needed to further investigate this relationship. In addition, this study exclusively utilized male mice. Although female mice are more resistant to the development of HG and demonstrate fewer behavioral changes (Kusumo et al. 2024), future studies are needed to investigate changes in negative valence bias within female hyperglycemic mice.

## Author Contributions

**Kayla R. Gilley‐Connor**: conceptualization, methodology, investigation, writing – original draft. **Laura E. Kusumo**: conceptualization, investigation, formal analysis, writing – review and editing. **Grace M. Hall**: conceptualization, methodology, investigation, writing – review and editing. **Matthew J. Folh**: investigation, writing – review and editing. **Elisabeth G. Vichaya**: conceptualization, supervision, funding acquisition, project administration, writing – original draft.

## Funding

Research reported in this publication was supported in part by the National Institute of Mental Health of the National Institutes of Health under award number R15MH139024. The content is solely the responsibility of the authors and does not necessarily represent the official view of the National Institutes of Health.

## Ethics Statement

The Baylor University Institutional Animal Care and Use Committee approved all animal procedures. The study was conducted, and the manuscript was prepared following the ARRIVE guidelines.

## Conflicts of Interest

The authors declare no conflicts of interest.

## Data Availability

The data file associated with this study will be made available via Baylor University's Digital Repository (BEARdocs).
